# New Materials for 3D-Printing Based on Polycaprolactone with Gum Rosin and Beeswax as Additives

**DOI:** 10.3390/polym12020334

**Published:** 2020-02-05

**Authors:** Cristina Pavon, Miguel Aldas, Juan López-Martínez, Santiago Ferrándiz

**Affiliations:** 1 Instituto de Tecnología de Materiales, Departamento de Ingeniería Mecánica y de Materiales, Universitat Politècnica de València, Plaza Ferrándiz y Carbonell s/n, 03801 Alcoi, Spain; jlopezm@mcm.upv.es (J.L.-M.); sferrand@mcm.upv.es (S.F.); 2 Departamento de Ciencia de Alimentos y Biotecnología, Facultad de Ingeniería Química y Agroindustria, Escuela Politécnica Nacional, 170517 Quito, Ecuador

**Keywords:** gum rosin, beeswax, 3D-printing, polycaprolactone, natural additives

## Abstract

In this work, different materials for three-dimensional (3D)-printing were studied, which based on polycaprolactone with two natural additives, gum rosin, and beeswax. During the 3D-printing process, the bed and extrusion temperatures of each formulation were established. After, the obtained materials were characterized by mechanical, thermal, and structural properties. The results showed that the formulation with containing polycaprolactone with a mixture of gum rosin and beeswax as additive behaved better during the 3D-printing process. Moreover, the miscibility and compatibility between the additives and the matrix were concluded through the thermal assessment. The mechanical characterization established that the addition of the mixture of gum rosin and beeswax provides greater tensile strength than those additives separately, facilitating 3D-printing. In contrast, the addition of beeswax increased the ductility of the material, which makes the 3D-printing processing difficult. Despite the fact that both natural additives had a plasticizing effect, the formulations containing gum rosin showed greater elongation at break. Finally, Fourier-Transform Infrared Spectroscopy assessment deduced that polycaprolactone interacts with the functional groups of the additives.

## 1. Introduction

Polymers are very versatile materials with a wide range of mechanical properties and high durability; therefore, these materials are produced in large volumes. Polymers have a wide variety of applications, including every-day utensils, clothing, packaging, engineering, and healthcare [1,2]. Nevertheless, because of their durability, polymers generate environmental issues leading to waste accumulations in landfills and oceans [1]. There is significant concern about the environmental impacts caused during the production process and their disposal, as most of the polymers are produced from non-renewable sources. According to The United Nations Environment Programme, 13 million tons of plastic waste are annually discharged into the ocean. If the production and consumption rates of plastic materials continue at the current levels, by 2050 there will be 12 million tons of plastic into nature and landfills [3]. Thus the formulation of new materials, less damaging and more environmentally friendly is of great interest, not only to reduce the use of traditional plastics, but also to replace their applications and uses [1,4].

Biopolymers have been recently considered as a suitable alternative as they are obtained or produced from biomass synthesis [5,6]. However, the use of biopolymers presents two main disadvantages. First, they are highly expensive as they are relatively new and the technology to produce them is more developed than those used in fossil-based polymers [7]. Secondly, biopolymers have lower mechanical properties than traditional polymers [4,8]. For these reasons, in general, most of the biopolymers are used as blends or fillers in the processing of traditional polymers [6,9,10,11].

In this work, polycaprolactone (PCL) was studied. Polycaprolactone is a biocompatible, biodegradable, and non-toxic biopolymer [12,13,14]. The thermal, physical, and mechanical properties depend on the degree of crystallinity. The degree of crystallinity varies with the molecular weight and has a maximum value of 69% in PCL [15,16]. PCL is highly ductile and flexible due to its low glass transition temperature (−60 °C) [17]. Additionally, PCL presents melting temperatures between 59 °C and 64 °C [18]. Moreover, PCL has good compatibility, and, it is usually blended with more rigid polymers in order to increase their flexibility and resistance to cracking. Also, it facilitates the coloration of polymers given its hydrophobicity [16,19]. Finally, research on the use of PCL in three-dimensional (3D)-printing has increased over the last 10 years. According to Web of Science (WOS), in 2009 only one article on this matter was published, while in 2019 more than 80 studies have been found. Consequently, 3D-printing was chosen to assess the material behaviour.

Gum rosin (GR) and Beeswax (BW) were chosen as natural additives. GR is the non-volatile component of pine resin and is mainly composed of resin acids. The structure of GR can be modified given its acidity and hydrophobicity [20,21,22,23]. GR has a glassy, translucent, and bright appearance, and a yellowish coloration [24,25]. Its melting temperature is around 75 °C [24,26]. In addition, gum rosin exhibits thermoplastic behaviour [27]. This material is fragile and rigid due to its polycyclic structure [28]. Additionally, gum rosin and its derivatives present antimicrobial activity [22], biodegradability, and biocompatibility [29,30]. It has low cost and availability [31]. Beeswax (BW) is an extremely ductile material, that is safe for human consumption, biodegradable, and biocompatible [32]. It is composed of at least 300 different compounds [33], that include alkanes, wax esters (mono-, di-, and hydroxylated esters), and free fatty acids [34,35]. Beeswax also presents a semi-crystalline structure with melting temperatures from 61 to 67 °C [33,36]. Gum rosin and beeswax are considered antagonistic materials due to their properties and behaviour. Nonetheless, Gaillard et al. (2011, 2012) conclude that, when the appropriate proportions are used, these materials can behave as complementary materials with synergistic effects [36,37].

Gum rosin has been blended with different polymeric matrices and the obtained materials have maintained its functional properties, as the antimicrobial activity [38,39], biocompatibility [40,41], and biodegradability [30]. The antimicrobial activity of BW was studied in synergy with other natural products [42]. The antimicrobial activity of PCL and gum rosin blends have been demonstrated in different ratios with enzymatic degradation [38]. Therefore, it is presumed that, when mixed, the resulting material will maintain the properties of all the components and enhance the antimicrobial properties. Additionally, BW has been proved to work well with synthetic polymers in drug delivery systems [32]. Furthermore, GR and BW mixtures improve adhesion, toughness [36], and plastic behaviour [37].

In this work, different blend formulations using PCL as the matrix, and gum rosin and beeswax as natural additives, were studied in order to produce environmentally friendly materials. All the formulations were first processed by melt-extrusion. Next, the bed and nozzle temperatures for each formulation were determined through a preliminary assessment. Then, the filaments were processed in a 3D printer to obtain samples for characterization. Finally, the physical, thermal, mechanical, and microstructural properties of each material were used to determine the effect of the additives on the PCL. Due to their biocompatibility and antimicrobial properties, as reported in the literature, these materials could be potentially suitable for biomedical applications using 3D-printing techniques, such as a surgical tools, medical devices, and prosthesis.

## 2. Materials and Methods

### 2.1. Materials

Polycaprolactone (PCL) Capa^TM^ 6800, commercial grade, was used as a matrix and was kindly supplied by Perstorp UK Ltd. (Warrington, UK). It has a density of 1.15 g/cm^3^ and melt flow index (MFI) of 2–4 g/10 min (160 °C, 2.16 kg). Gum rosin and beeswax were used as natural additives. Gum rosin (GR) was kindly supplied by Luresa Resinas S.L (Segovia, Spain). This resin has a softening point of 76 °C, an acidity index of 167 and a colour value of 4+ on the Gardner scale. The beeswax (BW) was acquired through a professional beekeeper from the Depósito Municipal de Abejas de Alcoy (Alcoy, Spain). Such beeswax has a melting temperature of 63 °C.

### 2.2. Filament Production and Characterization

The filaments for 3D-printing were formulated with 10 wt % as follows: (1) PCL with 10 wt % gum rosin, label as PCL-GR; (2) PCL with 10 wt % beeswax, label as PCL-BW; (3) PCL with 5 wt % gum rosin and 5 wt % beeswax, label as PCL-GR-BW. Additionally, neat PCL was assessed as a blank. The prepared formulations and the neat PCL were processed in a co-rotating twin-screw extruder Dupra S.L (Castalla, Spain) at 10 rpm, with a temperature profile of 40, 60, 70, and 80 °C (from hopper to die). The resulting material was milled into pellets. The pellets were processed in a filament extruder model EX2 from Filabot (Barre, PA, USA) to obtain the filament required for the 3D-printing process. The temperature used in the process was 80 °C with a 2.95 mm diameter nozzle for PCL, PCL-GR, and PCL-GR-BW; and at 90 °C with a 3.95 mm diameter nozzle for PCL-BW. The filaments were cooled down in a water bath. The diameter of the obtained filaments was constant between 2.80 and 3.00 mm.

The filaments (FS) were characterized by a mechanical tensile test according to ISO 527 standard test method. Cylindrical samples of 8 mm of length and 2.8–3.0 mm of diameter were used. The test was performed in a universal testing machine Elib 30 SAE Ibertest (Madrid, Spain). A 5 kN load cell and a crosshead rate of 50 mm/min were used in all test samples [43,44]. For each material, five samples were assessed. The average and the standard deviation of the measurements were reported. The statistical analysis was performed with OriginPro 8 software from OriginLab (Northampton, MA, USA). The significant differences were assessed at 95% confidence level according to Tukey’s test using a one-way analysis of variance (ANOVA).

### 2.3. Printability Tests and Rheology Characterization

The 3D-printing process was performed in a 3D printer BCN3D (Barcelona, Spain), with a 0.6 mm diameter nozzle. A rectilinear filling pattern was used for both the inner layers, as well as for the lower and upper layers, with 100% of filling. 52.2 cm of filament for each sample of each formulation were required.

The bed and nozzle temperatures were defined in printing trials, in order to obtain uniform printed samples. At first, the nozzle temperature was set at 80 °C, which was the endset temperature obtained from the Differential Scanning Calorimetry (DSC) curves. A visual examination was conducted to evaluate the quality of the printed objects. In those objects that presented structural defects, the nozzle temperature was gradually increased until there were no irregularities. The bed temperature was set at 40 °C for all the samples, while the nozzle temperature varied depending on the formulation as follows: 80 °C for PCL, 110 °C for PCL-GR, 90 °C for PCL-GR-BW, and 150 °C for PCL-BW.

Additionally, a parallel-plate oscillatory rheometry test was conducted in an AR-G2 rheometer from TA Instruments (New Castle, DE, USA) to study the rheological behaviour of the samples at printing temperatures. The test was conducted at two different temperatures: 80 °C (endset DSC temperature) and printing temperature previously selected on each formulation. The angular frequency varied between 100–0.01 rad/s and the maximum shear strain (γ) was set to 1%. The evolution of storage moduli (G’) as function of the angular frequency (ω) was reported. The storage modulus is linked to the ability of the material to hold its shape after the printing process [45].

### 2.4. Characterization of Standard Samples Obtained by the 3D-Printing Process

The filaments obtained previously were used to 3D print the standard samples according to ISO 527. The mechanical properties between the filaments (FS) and the standard test specimens (STS) were compared. The test parameters were the same used for the FS mechanical characterization.

### 2.5. Thermal Characterization

Differential Scanning Calorimetry (DSC) tests were carried out in a DSC 821 calorimeter from Mettler-Toledo (Schwerzenbach, Suiza). The used sample weight between 5 and 6 mg. The thermal cycle consisted of two heatings: a first heating from −50 to 120 °C, followed by a cooling step to −50 °C, and a second heating from −50 to 250 °C. The heating and cooling rate of all cycles were 10 °C/min and the tests were done in a nitrogen atmosphere with a flow rate of 30 mL/min. The melting temperature (*T*_m_) and the crystallization temperature (*T*_c_) were determined as the peak of the second heating, and the cooling curve, respectively.

The thermogravimetric analysis was conducted in a TGA PT1000 from Linseis (Selb, Germany), using a sample between 15 and 20 mg. The heating was carried out at a rate of 10 °C/min from 30 to 700 °C in a nitrogen atmosphere with a flow rate of 30 mL/min. The onset degradation temperature (*T*_5%_) was determined from the TGA curve at the temperature to which the material loses 5% of its initial mass. As well, the endset degradation temperature (*T*_90%_), in which the material loses 90% of its initial mass, was reported_._ Additionally, the maximum degradation rate temperature (*T*_max_) was determined at the peak of the first derivative of the TGA curve (DTG).

The Heat Deflection Temperature (HDT) was determined on a VICAT/HDT DEFLEX 687-A2, Metrotec SA (San Sebastián, Spain). The HDT was assessed according to the ISO 75 standard, following the A method, using a force of 3.2 N and a bath heating speed of 120 K/h. The dimensions of the STS samples were: 80 ± 2.0 × 10 ± 0.2 mm^2^ and 4 ± 0.2 mm thickness [46,47]. Five samples of each material were tested; the average and standard deviation of the measurements were reported. The statistical differences in the thermal properties were assessed by the one-way analysis of variance (ANOVA), at 95% confidence, as mentioned above.

### 2.6. Mechanical Characterization

The tensile characterization of the standard test specimens was performed under the same conditions used for the filament characterization (FS). Additionally, the flexural properties of the STS were determined according to the ISO 178 standard, using a 5 kN load cell and at a crosshead rate of 50 mm/min [43,44,48]. Five samples were tested for each formulation; the average and the standard deviation were reported.

Shore D hardness of the STS samples was determined using a durometer, model 673-D, S.A, Instruments J. (Barcelona, Spain), according to the ISO 868 standard. The measurements were taken in 20 different positions on each sample, having at least 6.0 mm separation among each measurement [49]. The average and the standard deviation were reported. All the obtained results were statistically analyzed in the same conditions, as previously reported.

### 2.7. Dynamic Mechanical Thermal Analysis

Dynamic Mechanical thermal analysis (DMTA) was carried out in DMA1 Mettler-Toledo (Schwerzenbach, Switzerland). The dimensions of the used samples were: 20 ± 2.0 × 4.5 ± 0.2 mm^2^ and 1 ± 0.2 mm of thickness. A single cantilever mode was used in the DMTA with a maximum deformation of 10 μm. The samples were exposed to a temperature sweep from −100 up to 40 °C with a frequency of 1 Hz, and the heating rate was set to 2 °C/min.

### 2.8. Fourier-Transform Infrared Spectroscopy—Attenuated Reflectance

The microstructure of the samples was assessed by a Fourier-Transform Infrared Spectroscopy analysis (FTIR), which was performed in an Spectrum BX FTIR equipment from Perkin-Elmer (Beaconsfield, UK) coupled with an ATR MlRacle ™ Pike Technologies (Madison, WI, USA). Every sample was examined in mid-infrared from 4000 to 600 cm^−1^, using 20 scans and 16 cm^−1^ of resolution.

### 2.9. Microstructural Characterization

Scanning electron microscopy (SEM) from the cryofractured surface of each sample was performed on a Phenon SEM equipment of FEI (Eindhoven, The Netherlands), with a voltage of 5 kV. Previously, the samples were coated with a gold-palladium alloy layer in a Sputter Mod Coater Emitech SC7620, Quorum Technologies (East Sussex, UK) to make the sample surface conductive.

### 2.10. Surface Characterization

Wettability was assessed by measuring the static contact angle of a droplet of distilled water on the surface of the samples. The contact angle measurements were determined using an optical goniometer EasyDrop-FM140 from Kruss Equipments (Hamburg, Germany) at room temperature. Additionally, the surface colour of each formulation was determined using a Colorflex-Diff2 458/08 colorimeter from HunterLab (Reston, VA, USA), under the CIE L*a*b* colour space. The L*, a*, and b* coordinates were reported. L* represents the brightness, and the parameters a* and b* show the intensity of the opposing colours green-red and blue-yellow, respectively [50,51]. Furthermore, the yellowing index (YI) and the total colour difference (ΔE) were reported. The ΔE is a numerical comparison of a sample’s colour to the standard (neat PCL) and was calculated using Equation (1) [9,50]:

(1) $$\Delta E\;=\;\sqrt{\Delta a^{*}{}^{2}+\Delta b^{*}{}^{2}+\Delta L^{*}{}^{2}}.$$

For wettability and colour characterization, 10 measurements on each sample were performed; the average and standard deviation were reported. While, the surface characterization data was statistically analyzed using the same parameters previously described.

## 3. Results and Discussion

### 3.1. Filament Production and Characterization, Printability Tests and Rheology Characterization

The values of the tensile strength for all the formulations showed significant differences (*p* < 0.05). GR, as well as BW, caused a decrease in the PCL mechanical strength. In the PCL-GR formulation, the average value of the tensile strength decreases by 28% with respect to the neat PCL. The PCL-BW decreased by 46.17%. However, in the PCL-GR-BW this value decreases only by 15%, since a synergistic effect of both additives in the mechanical strength was observed, due to their complementary characteristics [36]. Therefore, the extrusion of PCL-GR-BW results in a simpler process than PCL-GR and PCL-BW. The tensile strength values are important for 3D-printing processes, as they are related to the ease of traction of the material. Table 1 shows the 3D-printing parameters and the tensile properties of the obtained filament (FS) from PCL, and its formulations with GR and BW. Pictures of FS and STS are shown in Table 1.

Young’s modulus of the neat PCL was not statistically different from that of the PCL-GR. Meanwhile, the modulus decreased for the PCL-GR-BW and PCL-BW. The elongation at break compared to the neat PCL increased 63.27% in the PCL-GR, 21.12% in the PCL-GR-BW, and 31.71% in the PCL-BW. These results show that the addition of GR to the PCL not only gives plasticization to the structure but also increased in the toughness of the material. In relation to PCL-GR-BW and PCL-BW formulations, the material turns more ductile due to the plasticization effect [35,52].

**Figure 1:**
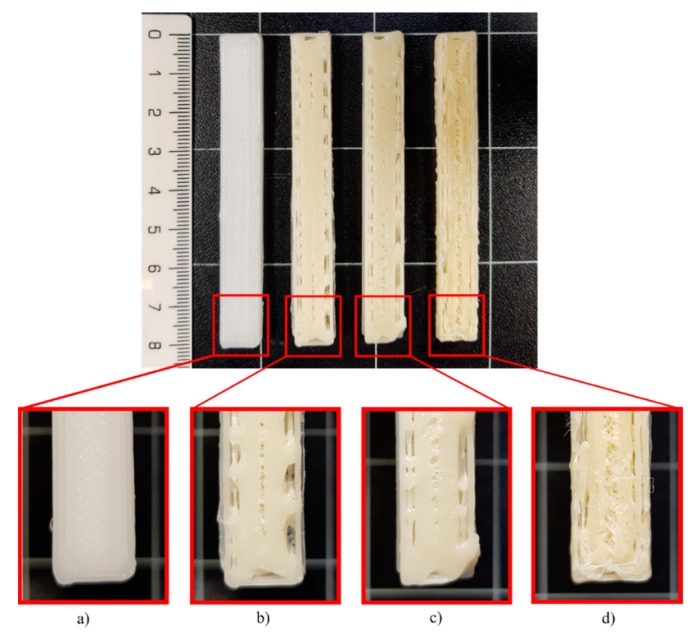
Standard test specimens (STS)surface obtained in the printing test at 80 °C for (**a**) PCL, (**b**) PCL-GR, (**c**) PCL-GR-BW and (**d**) PCL-BW.

All the studied materials used a bed temperature of 40 °C, meanwhile, the nozzle temperatures were different for all the formulations, being neat PCL the one with the lower values. These differences aim to achieve and adequate printability. Figure 1 shows the STS surface obtained after the printing tests for the studied materials at a nozzle temperature of 80 °C. For the neat PCL sample (Figure 1a), an object with a complete infill level and a homogeneous and smooth surface was obtained. In contrast, the printed samples from the other formulations (Figure 1b–d) were deformed, sagged, and show low infill levels. The neat PCL sample was easily extruded out from the nozzle at 80 °C. The defects in the other formulation were attributed to a broken extrusion thread, so that the extruded parts did not attain proper mechanical strength to support the following deposited layers, resulting in the collapse of the structures [53]. However, when the nozzle temperature of each formulation was increased, the resulting samples show high infill levels, good resolution and a homogeneous structure, as can be seen in the STS pictures in Table 1.

**Figure 2:**
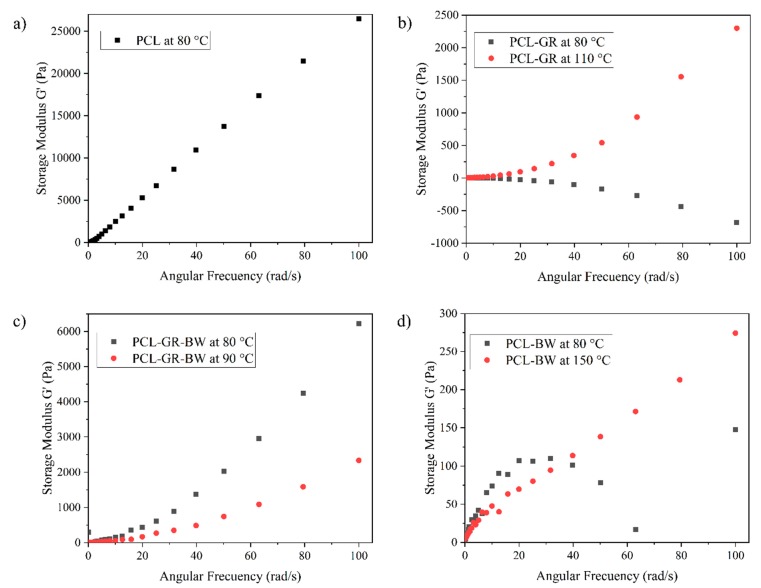
Storage modulus (G’) versus angular frequency (ω) for (**a**) PCL, (**b**) PCL-GR, (**c**) PCL-GR-BW and (**d**) PCL-BW.

As stated by Huang et al. (2018), in an extrusion-based 3D-printing, the storage modulus (G’) is related to the rheology of the material and it shows the material stiffness and ability to hold its shape after the 3D-printing process [45]. Figure 2 shows the storage modulus (G’) as function of the angular frequency (ω). G’ in PCL increases linearly with the frequency at 80 °C (Figure 2a). Meanwhile, when testing the materials that contain GR or BW, at 80 °C, G’ tend to decrease with the increment of the frequency (Figure 2b,d). This behaviour indicates that at 80 °C the PCL-BW and the PCL-GR formulations have lower resistance to the elastic deformation than neat PCL [54]. Therefore, in GR and BW formulations the extruded 3D-printed objects will not withstand the printed shape [55], as seen before in the printing tests results of Figure 1. However, when testing the same formulation at printing temperatures of 110 °C for PCL-GR and 150 °C for PCL-BW, G’ increases and turns directly dependent upon ω. Thus, an increment in the nozzle temperature helps to obtain a fused and cohesive material with better mechanical strength [54]. Consequently, 110 °C for PCL-GR and 150 °C for PCL-BW were chosen as the printing temperatures.

Figure 2c shows the PCL-GR-BW rheology assessment. As seen, at 80 °C, G’ increases with ω. This evidences the synergic effect between GR and BW when used together. GR provides rigidity, meanwhile, BW softness and ductility. Because some printing problems were detected at 80 °C, an increment of 10 °C in the nozzle temperature was considered. This increment turned the material more ductile (G’ trend decreases) with better printability behaviour.

### 3.2. Thermal Characterization

The matrix and the GR and BW additives have similar melting temperatures. Therefore, the DSC assessment will not show differences with the addition of additives. The melting temperatures (*T*_m_) between the formulations and neat PCL does not show statistical differences (*p* > 0.05), with an average value of 57.4 ± 0.6 °C. Moreover, the HDT values of both PCL-GR and PCL-BW do not have any statistical difference compared to PCL. On the contrary, the PCL-GR-BW formulation presents a significant decrease in the HDT value, probably due to the interaction of GR and BW. A similar HDT behaviour was attested by Aldas et al. (2019) when GR was used as an additive in a Mater-Bi^®^ bioplastic [56]. The DSC, HDT, and TGA assessment for the neat PCL and the studied formulations with GR and BW are summarized in Table 2.

**Figure 3:**
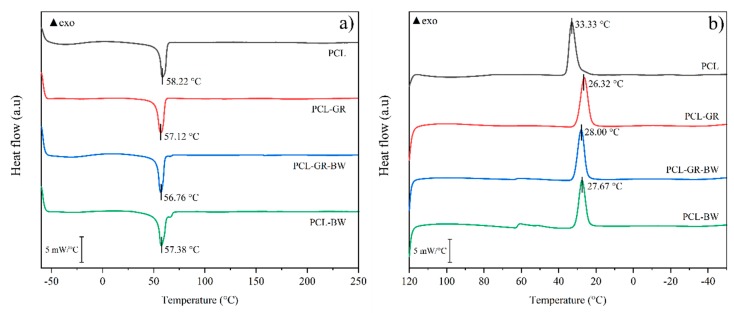
(**a**) DSC second heating curve and (**b**) DSC cooling curve of neat PCL and the formulations with GR and BW.

In Figure 3 the DSC heating and cooling curves are presented. The cooling curves show a significant decrease (*p* < 0.05) in the crystallization temperatures (*T*_c_), up to 7 °C, between the formulations and the PCL matrix. The natural additives increased the free volume on the PCL and, in consequence, the polymeric chains gained mobility. Hence, lower temperatures to recrystallize the PCL were needed when using those additives. All of the above implies a plasticizing effect of the additives [38].

**Figure 4:**
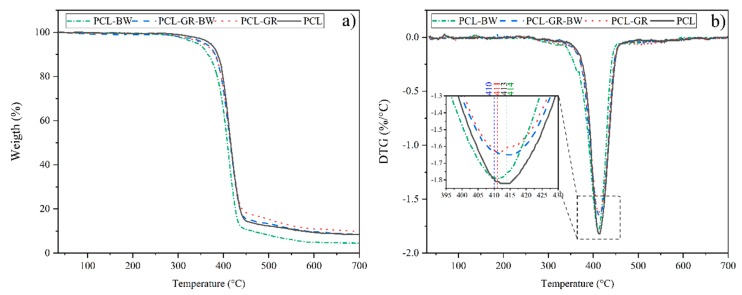
(**a**) TGA curves and (**b**) DTG curves with an expanded area for temperatures between 395 °C and 430 °C for the neat PCL and the formulations with GR and BW.

Figure 4 shows the TGA and DTG curves of neat PCL and the studied formulations. The TGA curves show that all the materials have three steps in thermal degradation. The first one is distinguished by a horizontal curve that indicates a constant mass of the sample. In the second step, starting from 300 °C, an abrupt change in the mass loss and an increment in the degradation rate was observed. Finally, at temperatures over 450 °C, small weight fluctuations with a tendency to become constant were detected [57].

With regard to the onset degradation temperature (*T*_5%_), significant differences (*p* < 0.05) were detected among the values for all the studied materials. The addition of GR decreased *T*_5%_ by 15 °C with respect to the PCL matrix. Such behaviour is in good accordance with the literature when using GR as an additive for biodegradable polymers [56]. *T*_5%_ decreased by 27 °C in PCL-BW, while on PCL-GR-BW only decreased 9 °C compared to the PCL. The decrement of the *T*_5%_ with respect to the matrix is mainly due to the low thermal stability of the natural additives [11]. No significant differences (*p* > 0.05) were found between the *T*_max_ values (expanded area in Figure 4) in the second step of the decomposition. The most noticeable difference in thermal stability was found in the third step of degradation. *T*_90%_ increased by 100 °C when only GR was added to the matrix (PCL-GR). In contrast, the addition of BW to the matrix (PCL-BW) caused a decrease of 120 °C, compared to neat PCL. Meanwhile, the addition of GR-BW does not show significant differences (*p* > 0.05) on *T*_90%_ compared to the matrix. The increase of thermal stability reached by PCL-GR contradicts the results obtained by Chang et al. (2018), who studied different blends of PCL with GR. In that study, the addition of GR reduces the PCL thermal stability from 450 to 420 °C. This difference could be attributed to the methodology used to obtain the formulations. In the present study, an extrusion process was employed, while Chang et al. prepared the blends by melt mixing [38]. BW, on the other hand, reduced thermal stability due to the inherent thermal characteristics of this natural additive. In PCL-GR-BW both effects were balanced. Furthermore, it was possible to verify the miscibility between the additives and the PCL matrix with the TGA assessment, since no additional inflections were detected in the curves [57,58].

### 3.3. Mechanical Characterization

The tensile, flexural, and hardness properties of the STS are reported in Table 3. It was found that Young’s modulus decreased in all the studied formulations when compared with the neat PCL. These results show that PCL becomes more ductile with the incorporation of natural additives. This behaviour is in accordance with the thermal characterization data, which showed that BW and GR have a plasticization effect on the PCL structure [11]. Consequently, GR and BW reduce the interactions of the PCL molecules and allow a free movement of the chains causing a reduction in Young’s moduli values [59]. The statistical analysis showed significant differences between the PCL and the formulations moduli (*p* < 0.05). Nevertheless, Young’s moduli of PCL-GR and PCL-GR-BW have not significant differences (*p* > 0.05). As PCL-BW exhibits the lower Young’s modulus value among the tested materials, it can be considered that BW provides higher ductility than GR.

Significant differences (*p* < 0.05) were found in the elongation at break values (Table 3), between PCL, PCL-GR, and PCL-BW. The elongation at break value of neat PCL increases 30% with the addition of GR, while the addition of BW decreases it in 50%. Whereas, PCL-GR-BW does not show significant changes (*p* > 0.05). The relation between Young’s Modulus along with the elongation at break indicates that GR acts as a plasticizer in the PCL structure. As stated by Narayanan et al. (2017), this additive improves the ductility, as well as the elongation at the break of the material [11,56]. In contrast, the addition of BW, not only provides more ductility to PCL, but also reduced its elasticity, causing a fall in the toughness of the material [35,52]. The addition of GR, BW, and GR-BW produced a significant decrease (*p* < 0.05) in the tensile strength of neat PCL, due to the increase in the ductility of the PCL structure [60].

The decrease in the flexural and hardness properties of the formulations (Table 3) confirms the plasticizing effect produced by the natural additives in the PCL structure [52]. PCL-GR and PCL-GR-BW do not present significant differences (*p* > 0.05) in flexural properties. The flexural modulus in both materials decreases by 48%, while the maximum flexural resistance decreases by 13%, compared to the neat PCL. The PCL-BW flexural modulus is the lowest one, which confirms that BW reduced the mechanical properties because of its softness. The PCL-BW maximum resistance is statistically equal to the PCL-GR. This indicates that the rigidity of GR and the ductility of BW produced a similar effect in the flexural resistance of the formulations. PCL-GR and PCL-GR-BW hardness present no significant differences (*p* > 0.05). In both materials, the hardness is 12% lower than PCL hardness. PCL-BW hardness presents significant differences (*p* < 0.05) with a decrease in 23% compared to the matrix.

The mechanical properties confirm that GR helps to prevent the loss of the properties of the matrix. PCL-GR-BW values are statistically equal to those of PCL-GR [61]. As stated by Chang et al. (2018), when 10% of GR is added to a PCL matrix, this additive acts as a nucleating agent. As a result, the material becomes more rigid and its mechanical characteristics are unaffected [38]. On the other hand, the addition of BW provided a major ductility and reduced mechanical properties.

**Figure 5:**
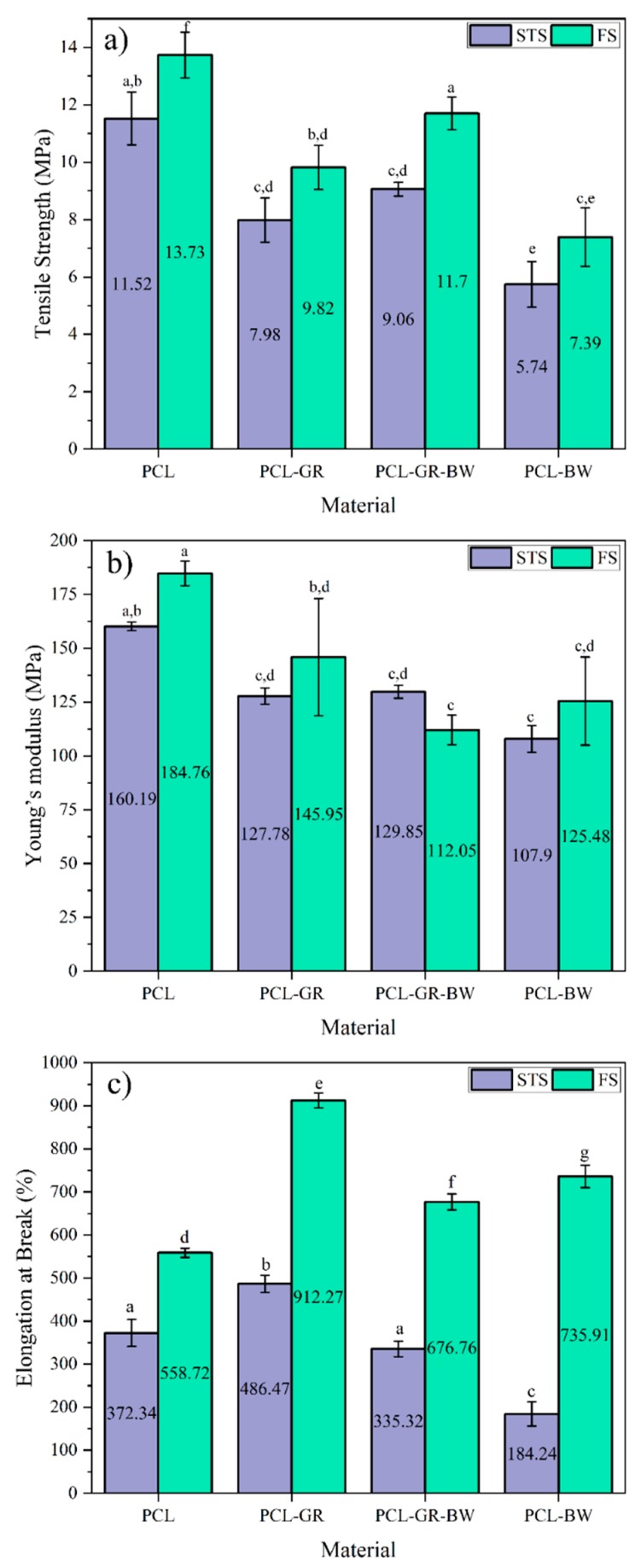
Comparison of (**a**) tensile strength, (**b**) Young’s Modulus, and (**c**) elongation at Break of STS (standard test specimen) and FS (filament samples) of neat PCL and the formulations with GR and BW filaments. ^a–g^ Different letters within the same property show statistically significant differences between formulations (*p* < 0.05).

A comparison among the mechanical tensile properties of STS and FS is presented in Figure 5. The tensile strength values (Figure 5a) in all the formulation decrease respect to neat PCL, both in FS and STS. In FS the values of PCL-GR and PCL-GR-BW are statistically equal (*p* > 0.05). Meanwhile, in the STS, PCL-GR-BW tensile strength is statistically higher than PCL-GR. The differences between FS and STS tensile strength could be attributed to several factors, including the chain orientation given to the material during the extruding process, the geometry of the samples, and the additional thermal process that the STS were subject to [62]. PCL-GR-BW presents the highest tensile strength value among all the formulations, both in FS and STS. This shows that the interaction between GR and BW helps the material not lose its mechanical tensile properties. In this context, BW improves the toughness of the material, while the rigidity of GR helps to maintain its mechanical properties [21,36,37].

Additionally, Young’s moduli of all the formulated materials decreased, compared to the matrix (Figure 5b). The greatest decrease was determined in PCL-GR-BW in the filament shape (FS). This reduction could be attributed to the effect of the GR-BW mixture that provided more ductility to the material. This confirms that the additives cause a reduction in PCL strength, and consequently, an increase in the ductility of the material. Moreover, the formulations in Young’s moduli were statistically equal (*p* > 0.05).

The elongation at break of all the studied materials is presented in Figure 5c. It can be observed FS values were higher than the values achieved for the STS. This difference is attributed to the process used to obtain the samples. While the FS was obtained from a direct extrusion of the material, the STS was obtained after a 3D-printing process. According to Eshraghi and Das (2010), the mechanic characteristics of PCL depend on the orientation that the chains adopt during the processing of the material. Therefore, an increase in the elongation at break is obtained when the polymer chains acquire a longitudinal position, as the case of the filaments (FS) [62]. Furthermore, in the FS samples, the addition of the natural additives contributes to enhance the elongation at break values, compared to the neat PCL. Therefore, the plasticization of PCL due to the addition of GR, BW, or GR-BW is confirmed in the case of the filaments. The plasticization shows a decrease in the tensile strength and an increase in the elongation at break of the material. [60]. In the STS values, the addition of GR increases the elongation at break of the PCL-GR, while PCL-GR-BW did not show significant differences in this property. Nevertheless, the addition of BW in the STS decreases the elongation at break, which could indicate a poor interaction between BW and PCL after the 3D-printing process.

### 3.4. Dinamic Mechanical Thermal Analysis

**Figure 6:**
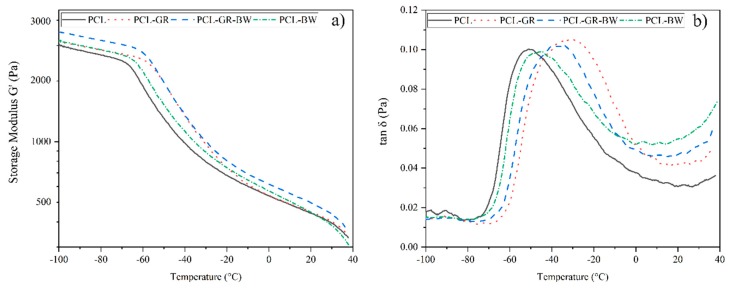
(**a**) storage modulus and (**b**) loss factor from DMTA analysis for PCL and its formulations with GR and BW.

The evolution of the storage modulus (G’) and the loss factor (tan δ) against the temperature is plotted in Figure 6. In the formulated materials, it is observed an increment of G’ for temperatures below 0 °C, due to the the presence of the additives. For temperatures above 0 °C, the G’ of the formulated materials (PCL-GR, PCL-GR-BW and PCL-BW) tends to the modulus of PCL. The rigidization of the structures in low temperatures could be attributed to the freezing and crystallization of the GR and BW large compounds. GR is composed mainly of hydrophenanthrene rings and BW is composed of lipids. Therefore, when cooled, they could act as reinforcements or fillers [1,2]. Thus, a reduction in temperature restricts the movement of the PCL chains, producing higher values of G’ when GR or BW are added.

In the loss factor curves (Figure 6b), it is possible to determine the glass transition temperature (*T*_g_) of PCL at −50.0 °C. After the addition of the natural additives, a shift of this peak to higher temperatures is detected. The corresponding *T*_g_ are located at −44.5, −37.3 and −30 °C, for PCL-BW, PCL-GR-BW, and PCL-GR, respectively. Once again, this behaviour shows a rigidization of the structure due to the additives. Importantly, for temperatures above 0 °C, the additives act as plasticizers, thereby, reducing the Young’s modulus and increasing the elongation at break in the formulations, as discussed in the mechanical characterization. The presence of a sole peak in the loss factor curves comfirms a thermodynamic compatibility between PCL and the additives. This behaviour is in good agreement with the results obtained from DSC and TGA analyses [3,4,5].

### 3.5. Fourier Transform Infrared Spectrometry Assessment

**Figure 7:**
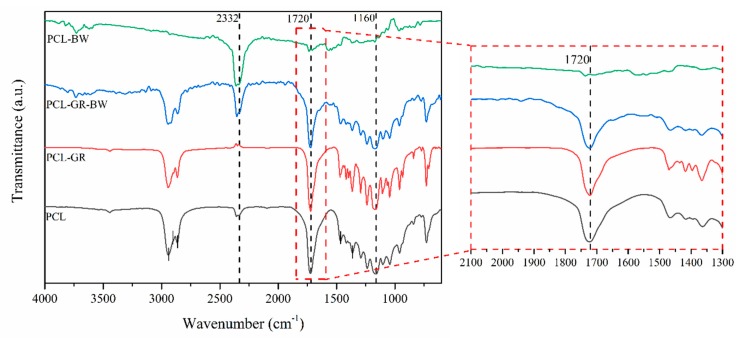
Fourier-transform infrared spectroscopy (FTIR) spectra of neat PCL and the formulations with GR and BW with an expanded area for ranges between 2100 and 1300 cm^−1^.

Figure 7 shows the FTIR spectra of the studied formulations and the neat PCL. On the PCL spectrum, the characteristic band, corresponding to the vibrations and extensions of the C–H groups, can be observed at 2940 and 2860 cm^−1^. Further, the bands corresponding to the C=O and C–O–C groups were found at 1720 and 1160 cm^−1^. In addition, the representative bands of the methylene group (–CH_2_) were located at 1470 and 1358 cm^−1^ [38].

The literature refers that the characteristic bands of the acid dimers, the C=O stretching and C–O–C linkages of the carboxylic acids, fatty acids, phospholipids, and triacylglycerols of GR can be found at 1698 and 2910 cm^−1^ [38,57,63,64]. However, in the PCL-GR spectrum, overlap was detected between these bands and the ones in the matrix. This overlapping effect was also detected in the PCL-GR-BW.

The literature mentions that the representative band corresponding to methylene (–CH_2_–) and methyl (–CH_3_) groups should be found at 2916 and 2848 cm^−1^, and those corresponding to the vibrations of the mentioned groups at 1743 and 1170 cm^−1^ [65]. Nevertheless, in the PCL-BW spectrum, only small bands at 1743 and 1170 cm^−1^ were detected, possibly due to the low interaction between the matrix and the additive. Further, it is important to notice that the additives were added to the PCL in a 10 wt % concentration. Therefore, their characteristic bands are less intense than those of the matrix.

Finally, a reduction of the band area located at 1720 cm^−1^ was identified when GR was added to the matrix (expanded area Figure 7). This band corresponds to the C=O and C–O–C groups, and the area reduction implies that the addition of GR or GR-BW produced an interaction among the functional groups of the involved materials [38]. Nonetheless, in the PCL-BW, this characteristic band was not detected, since the BW does not interact with the PCL matrix. The absence of interaction is related to the poor mechanical properties of PCL-BW, as already discussed.

### 3.6. Microstructural Characterization

**Figure 8:**
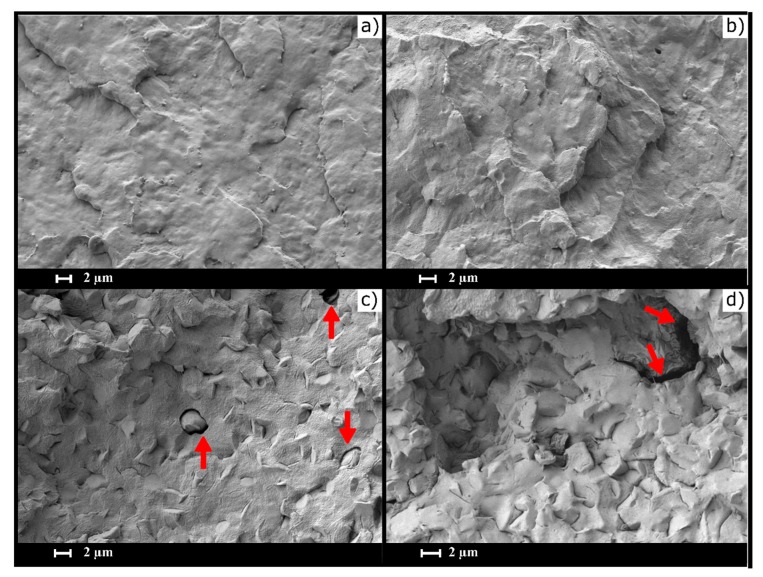
Scanning electron microscopy (SEM) images of (**a**) PCL, (**b**) PCL-GR, (**c**) PCL-GR-BW, and (**d**) PCL-BW, red arrows show holes and discontinuities in the material surface.

The scanning electron microscopy (SEM) images of the cryofractured surface are shown in Figure 8. As may be seen, comparing PCL (Figure 8a) and PCL-GR (Figure 8b), the addition of GR did not cause any significant change in the morphology of the polymer surface. Hence, the surface in PCL and PCL-GR were homogeneous and smooth with no visible phase separation. This homogeneous morphology is due to the affinity of GR with the PCL which enhances the compatibility of the materials [66]. Accordingly, the homogeneous distribution of GR in the matrix increased the miscibility of both components [56,67]. These SEM images agree with the results of Chang et al. (2018), who made different formulations of PCL with pine resin, and determined that the morphology of the formulations was similar to that of neat PCL [38].

On the other side, the materials formulated with BW exhibited two visible phases in the fracture surface (Figure 8c,d), being the disperse phase the one corresponding to the BW. Irregular structures were embedded in the matrix phase and were found in greater proportion in the PCL-BW, which has a higher content of the additive. According to Fabra et al. (2019), the presence of lipids leads to structures with irregular topographies [68].

Notably, the BW structures in the PCL-BW reach 4 µm, while the PCL-GR-BW reaches 2 µm. The reduction of the BW structures size shows that GR helps to disperse BW in the matrix. Finally, a bad phase cohesion was observed in both formulations. The bad phase cohesion can be seen as holes and discontinuities in the surface of the materials (red arrows). This effect is related to low miscibility among the components of the formulations [69], and it is less noticeable in the PCL-GR-BW, where BW structures are more dispersed. Therefore, the mechanical properties of PCL-GR-BW were higher than those of PCL-BW, as discussed before.

### 3.7. Surface Characterizzation

The contact angle and CIEL*a*b* space values are shown in Table 4. The contact angle values were higher than 65°, therefore, all the studied materials present a hydrophobic behaviour [70]. The detected hydrophobicity is related to both PCL and the additives hydrophobic nature [19,38,42]. The wettability statistical analyses (Table 4) showed significant differences between all the formulations (*p* < 0.05). Moreover, the addition of GR, GR-BW, or BW increases the hydrophobicity of PCL in 6%, 26%, and 30%, respectively. According to Hambleton et al. (2009), this increment is due to the additives reducing the interactions between the polymer chains and increase its mobility. Hence, the additives on excess were brought to the surface and result in a hydrophobic behaviour [71,72].

The colour values of the samples (Table 4) present significant differences (*p* < 0.05) in the a* and b* parameters of all the studied materials. The a* coordinate values fluctuate around 0, which shows that neither the green nor the red colours are dominant in the materials. Additionally, the positive values of b* coordinate confirm yellow coloration in the formulations, with respect to neat PCL. The PCL-BW is the formulation with the highest yellow coloration (b* = 20.9). These results show that the natural colour of the additives directly influences the final coloration of the materials [51]. The L* between the neat PCL and PCL-GR-BW are statistically equal with an average value of 77.8 ± 0.8. The L* PCL-GR and PCL-BW with an average value of 96.4 ± 1.3 are also statistically equal (*p* > 0.05). This means that all the studied materials have a bright hue [50].

**Figure 9:**
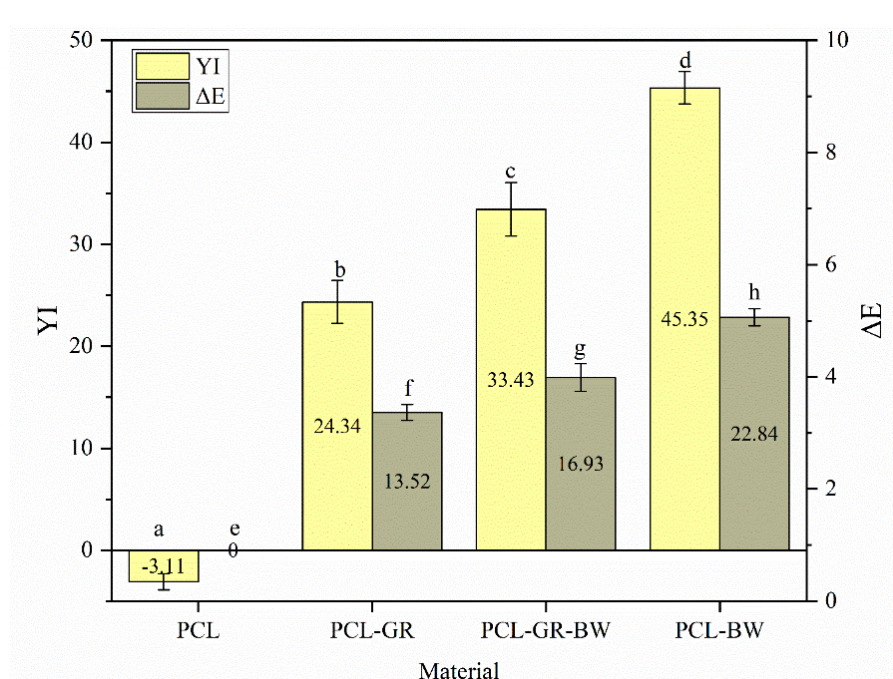
Total colour differences (ΔE) as well as changes in yellowness index (YI). ^a–h^ Different letters show statistically significant differences between formulations (*p* < 0.05).

Moreover, the yellowness index values (YI) and the total difference of colour (ΔE) of the studied samples are shown in Figure 9. The YI increased in those materials in which BW was added because of the intrinsic yellow coloration of the additive. All the ΔE values show a difference of more than two units. Therefore, the change in colour is appreciable to the human eye [71]. Significant differences (*p* < 0.05) among all the studied samples were determined.

## 4. Conclusions

The filaments for 3D-printing were successfully produced using a polycaprolactone as a matrix, and gum rosin and beeswax as natural additives. The parameters for the 3D-printing process were defined, resulting in a bed temperature of 40 °C for all the formulations, whereas the nozzle temperature varied between 90 °C and 150 °C, depending on the easiness of traction of the materials in the printer. The DSC assessment showed that all the natural additives acted as plasticizers, resulting in lower crystallization temperatures. Additionally, the thermal characterization by the TGA analysis helped to determine good miscibility between GR and BW with the PCL matrix. Moreover, it was concluded that GR helps to improve PCL thermal stability. On the other hand, BW decreased it, while the mixture GR-BW did not significantly change the thermal stability of the matrix. The mechanical characterization in the filaments showed that GR plasticize the PCL structure since its elongation at break is enhanced by 63%. The materials modified with BW and GR-BW exhibit low elongation at break (31% and 21%, respectively) but high ductility as the tensile strength decreased. The SEM characterization showed that when using GR as an additive, the material is composed of one phase; when using BW, the material exhibits two phases, resulting in low miscibility of the components and low mechanical properties. Colour measurements showed that the intrinsic colouration of natural additives has a significant effect on the colour of the final materials. With respect to wettability, the addition of GR and BW increased the hydrophobic behaviour of neat PCL. Finally, it was concluded that the PCL-GR-BW formulation is the most suitable material for a 3D-printing process as it behaves better in the traction mechanism of the printer. Further, it exhibits the thermal and mechanical properties closer to neat PCL.

## Figures and Tables

**Table 1:** Three-dimensional (3D)-printing parameters and tensile mechanical properties of filaments of neat polycaprolactone (PCL) and the formulations with gum rosin (GR) and beeswax (BW).

Material	PCL	PCL-GR	PCL-GR-BW	PCL-BW
**Bed Temperature (°C)**	40	40	40	40
**Nozzle Temperature (°C)**	80	110	90	150
**Filament**	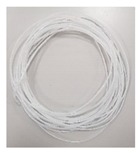	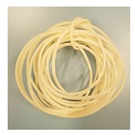	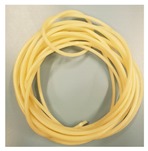	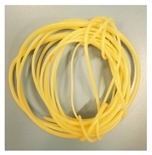
**Standard Test Specimens** **(STS)**	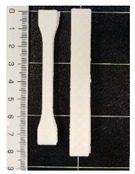	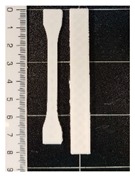	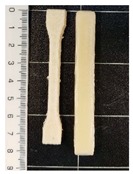	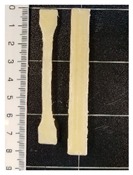
**Young’s Modulus (MPa)**	184.76 ± 5.70 ^a^	145.95 ± 27.23 ^a,b^	112.05 ± 6.87 ^b^	125.48 ± 20.45 ^b^
**Elongation at Break (%)**	558.72 ± 10.54 ^a^	912.27 ± 17.42 ^b^	676.76 ± 18.48 ^c^	735.91 ± 26.13 ^d^
**Tensile Strength (MPa)**	13.73 ± 0.80 ^a^	9.82 ± 0.77 ^b^	11.7 ± 0.57 ^c^	7.39 ± 1.02 ^d^

^a–d^ Different letters within the same property show statistically significant differences between formulations (*p* < 0.05).

**Table 2:** Thermal properties of neat PCL and its formulations with GR and BW.

Material	DSC		HDT	TGA		
	*T*_m_ (°C)	*T*_c_ (°C)	*T* (°C)	*T*_5%_ (°C)	*T*_90%_ (°C)	*T*_máx_ (°C)
PCL	58.2 ± 0.5 ^a^	33.3 ± 1.3 ^a^	43.6 ± 1.7 ^a^	369.8 ± 1.9 ^a^	588.0 ± 1.5 ^a^	413.0 ± 1.0 ^a,b^
PCL-GR	57.1 ± 0.4 ^a^	26.3 ± 1.4 ^b^	42.1 ± 1.3 ^a^	354.9 ± 2.1 ^b^	684.9 ± 0.3 ^b^	411.0 ± 1.4 ^a,b^
PCL-GR-BW	56.8 ± 0.6 ^a^	28.0 ± 0.8 ^b^	38.8 ± 0.9 ^b^	360.4 ± 0.8 ^c^	587.6 ± 0.7 ^a^	414.0 ± 1.3 ^b^
PCL-BW	57.4 ± 0.8 ^a^	26.7 ± 1.6 ^b^	41.5 ± 1.0 ^a^	342.7 ± 1.1 ^d^	463.9 ± 1.2 ^c^	410.0 ± 1.3 ^a^

^a–d^ Different letters within the same property show statistically significant differences between formulations (*p* < 0.05).

**Table 3:** Tensile, flexural and hardness properties of neat PCL and the formulations with GR and BW.

Material	Tensile Properties			Flexural Properties		Hardness
	Tensile Strength (MPa)	Elongation at BREAK (%)	Young’s Modulus (MPa)	Maximum Resistance (MPa)	Flexural Modulus (MPa)	Shore D
PCL	11.52 ± 0.65 ^a^	372.34 ± 31.37 ^a^	160.19 ± 2.01 ^a^	18.03 ± 1.15 ^a^	409.58 ± 12.73 ^a^	53 ± 1.64 ^a^
PCL-GR	7.98 ± 0.77 ^b^	486.47 ± 14.12 ^b^	127.78 ± 3.82 ^b^	15.67 ± 1.7 ^a,b^	231.35 ± 15.59 ^b^	47± 1.69 ^b^
PCL-GR-BW	9.06 ± 0.17 ^b^	335.32 ± 18.24 ^a^	129.85 ± 2.15 ^b^	17.00 ± 1.34 ^a^	211.20 ± 13.30 ^b^	46 ± 1.63 ^b^
PCL-BW	5.74 ± 0.8 ^c^	184.24 ± 28.26 ^c^	107.90 ± 6.19 ^c^	8.60 ± 1.06 ^b^	164.56 ± 17.21 ^c^	41 ± 2.39 ^c^

^a–d^ Different letters within the same property show statistically significant differences between formulations (*p* < 0.05).

**Table 4:** Wettability and colour parameters for the CIEL*a*b* space of neat PCL and the formulations with GR and BW.

Material	Wettability	Colour		
	Contact Angle (θ°)	L*	a*	b*
PCL	75.6 ± 1.0 ^a^	76.17 ± 0.40 ^a^	−0.92 ± 0.18 ^a^	−1.25 ± 0.32 ^a^
PCL-GR	80.0 ± 0.6 ^b^	70.74 ± 1.41 ^b^	−2.16 ± 0.10 ^b^	10.98 ± 1.05 ^b^
PCL-GR-BW	95.1 ± 1.4 ^c^	75.41 ± 0.69 ^a^	−1.33 ± 0.11 ^c^	15.65 ± 1.38 ^c^
PCL-BW	97.7 ± 1.1 ^d^	70.73 ± 0.83 ^b^	0.07 ± 0.04 ^d^	20.90 ± 0.81 ^d^

^a–d^ Different letters within the same property show statistically significant differences between formulations (*p* < 0.05).

## References

[1] Zhu Y., Romain C., Williams C.K. (2016). Sustainable Polymers from Renewable Resources. Nature.

[2] Aldas M., Paladines A., Valle V., Pazmiño M., Quiroz F. (2018). Effect of the Prodegradant-Additive Plastics Incorporated on the Polyethylene Recycling. Int. J. Polym. Sci..

[3] Our Planet Is Drowning in Plastic Pollution. https://www.unenvironment.org/interactive/beat-plastic-pollution/.

[4] Rudin A., Choi P. (2013). Biopolymers. The Elements of Polymer Science & Engineering.

[5] McKeen L. (2012). Renewable Resource and Biodegradable Polymers. The Effect of Sterilization on Plastics and Elastomers.

[6] Queiroz A.U.B., Collares-Queiroz F.P. (2009). Innovation and Industrial Trends in Bioplastics. Polym. Rev..

[7] Johnson M., Tucker N., Barnes S., Kirwan K. (2005). Improvement of the Impact Performance of a Starch Based Biopolymer via the Incorporation of Miscanthus Giganteus Fibres. Ind. Crops Prod..

[8] Lagaron J.M., Lopez-Rubio A. (2011). Nanotechnology for Bioplastics: Opportunities, Challenges and Strategies. Trends Food Sci. Technol..

[9] Arrieta M.P., Samper M.D., Jiménez-López M., Aldas M., López J. (2017). Combined Effect of Linseed Oil and Gum Rosin as Natural Additives for PVC. Ind. Crops Prod..

[10] Wilbon P.A., Chu F., Tang C. (2013). Progress in Renewable Polymers from Natural Terpenes, Terpenoids, and Rosin. Macromol. Rapid Commun..

[11] Narayanan M., Loganathan S., Valapa R.B., Thomas S., Varghese T.O., Babu R., Thomas S., Varghese T.O. (2017). UV Protective Poly (Lactic Acid)/Rosin Films for Sustainable Packaging. Int. J. Biol. Macromol..

[12] Rudnik E. (2012). Compostable Polymer Properties and Packaging Applications. Plastic Films in Food Packaging: Materials, Technology and Applications.

[13] Lopresti F., Maio A., Botta L., Scaffaro R. (2016). Preparation and Mechanical Characterization of Polycaprolactone/Graphene Oxide Biocomposite Nanofibers. AIP Conf. Proc..

[14] Kouparitsas I.K., Mele E., Ronca S. (2019). Synthesis and Electrospinning of Polycaprolactone from an Aluminium-Based Catalyst: Influence of the Ancillary Ligand and Initiators on Catalytic Efficiency and Fibre Structure. Polymers.

[15] Labet M., Thielemans W. (2009). Synthesis of Polycaprolactone: A Review. Chem. Soc. Rev..

[16] Guarino V., Gentile G., Sorrentino L., Ambrosio L. (2017). Polycaprolactone: Synthesis, Properties, and Applications.

[17] McKeen L. (2012). Introduction to the Physical, Mechanical, and Thermal Properties of Plastics and Elastomers. The Effect of Sterilization Methods on Plastics and Elastomers.

[18] Woodruff M.A., Hutmacher D.W. (2010). The Return of a Forgotten Polymer—Polycaprolactone in the 21st Century. Prog. Polym. Sci..

[19] Zhang X., Peng X., Zhang S.W. (2016). Synthetic Biodegradable Medical Polymers: Polymer Blends. Science and Principles of Biodegradable and Bioresorbable Medical Polymers: Materials and Properties.

[20] Yao K., Tang C. (2013). Controlled Polymerization of Next-Generation Renewable Monomers and Beyond. Macromolecules.

[21] Mitchell G.R., Mahendra V., Sousa D. (2018). Biopolymers Based on Rosin. Curr. Res. Biopolym..

[22] Termentzi A., Fokialakis N., Leandros Skaltsounis A. (2011). Natural Resins and Bioactive Natural Products Thereof as Potential Antimicrobial Agents. Curr. Pharm. Des..

[23] Savluchinske-Feio S., Curto M.J.M., Gigante B., Roseiro J.C. (2006). Antimicrobial Activity of Resin Acid Derivatives. Appl. Microbiol. Biotechnol..

[24] Abdel-raouf M.E., Chem B., Abdel-raouf M.E., Abdul-raheim A.M. (2018). Rosin: Chemistry, Derivatives, and Applications: A Review. BAOJ Chem. Manar.

[25] Yadav B.K., Gidwani B., Vyas A. (2016). Rosin: Recent Advances and Potential Applications in Novel Drug Delivery System. J. Bioact. Compat. Polym..

[26] Maiti S., Ray S.S., Kundu A.K. (1989). Rosin: A Renewable Resource for Polymers and Polymer Chemicals. Prog. Polym. Sci..

[27] Kumar S., Gupta S.K. (2013). Rosin: A Naturally Derived Excipient in Drug Delivery Systems. Polim. Med..

[28] Huang W., Diao K., Tan X., Lei F., Jiang J., Goodman B.A., Ma Y., Liu S. (2019). Mechanisms of Adsorption of Heavy Metal Cations from Waters by an Amino Bio-Based Resin Derived from Rosin. Polymers.

[29] Schmitt H., Guidez A., Prashantha K., Soulestin J., Lacrampe M.F., Krawczak P. (2015). Studies on the Effect of Storage Time and Plasticizers on the Structural Variations in Thermoplastic Starch. Carbohydr. Polym..

[30] Satturwar P.M., Fulzele S.V., Dorle A.K. (2003). Biodegradation and in Vivo Biocompatibility of Rosin: A Natural Film-Forming Polymer. AAPS PharmSciTech.

[31] Gutierrez J., Tercjak A., Martin M.D., Tercjak A., Kortaberria G., de la Caba K., Riccardi C.C., Mondragon I., Chu F., Tang C. (2014). Natural Gum Rosin Thin Films Nanopatterned by Poly(Styrene)-Block-Poly(4-Vinylpiridine) Block Copolymer J. RSC Adv..

[32] Subha V., Arulsha W., Kirubanandan S., Renganathan S. (2018). Sustained Drug Delivery of Capecitabine Using Natural (Bee Wax) and Synthetic Polymer (PLGA). MOJ Drug Des. Dev. Ther..

[33] Tulloch A.P. (2015). Beeswax—Composition and Analysis. Bee World.

[34] Buchwald R., Breed M., Greenberg A., Otis G. (2006). Interspecific Variation in Beeswax as a Biological Construction Material. J. Exp. Biol..

[35] Morgan J., Townley S., Kemble G., Smith R. (2002). Measurement of Physical and Mechanical Properties of Beeswax. Mater. Sci. Technol..

[36] Gaillard Y., Mija A., Burr A., Darque-Ceretti E., Felder E., Sbirrazzuoli N. (2011). Green Material Composites from Renewable Resources: Polymorphic Transitions and Phase Diagram of Beeswax/Rosin Resin. Thermochim. Acta.

[37] Gaillard Y., Girard M., Monge G., Burr A., Ceretti E.D., Felder E. (2012). Superplastic Behavior of Rosin/Beeswax Blends at Room Temperature. J. Appl. Polym. Sci..

[38] Chang R., Rohindra D., Lata R., Kuboyama K., Ougizawa T. (2018). Development of Poly(ε-Caprolactone)/Pine Resin Blends: Study of Thermal, Mechanical, and Antimicrobial Properties. Polym. Eng. Sci..

[39] Moustafa H., El Kissi N., Abou-Kandil A.I., Abdel-Aziz M.S., Dufresne A., El Kissi N., Abou-Kandil A.I., Abdel-Aziz M.S., Dufresne A. (2017). PLA/PBAT Bionanocomposites with Antimicrobial Natural Rosin for Green Packaging. ACS Appl. Mater. Interfaces.

[40] Geurtsen W. (2000). Biocompatibility of Resin-Modified Filling Materials. Crit. Rev. Oral Biol. Med..

[41] Mahendra V. (2015). Fabrication of Biocompatible Hydrogels from Pine Resin.

[42] Fratini F., Cilia G., Turchi B., Felicioli A. (2016). Beeswax: A Minireview of Its Antimicrobial Activity and Its Application in Medicine. Asian Pac. J. Trop. Med..

[43] International Standards Organization (2012). ISO 527-1—Plastics—Determination of Tensile Properties—Part 1: General Principles.

[44] International Standards Organization (2012). ISO 527-2. Plastics—Determination of Tensile Properties—Part 2: Test Conditions for Moulding and Extrusion Plastics.

[45] Huang C.Y. (2018). Extrusion-Based 3D Printing and Characterization of Edible Materials. Master’s Thesis.

[46] International Standards Organization (2013). ISO 75-1:2013—Plastics—Determination of Temperature of Deflection under Load—Part 1: General Test Method.

[47] International Standards Organization (2013). ISO 75-2:2013—Plastics—Determination of Temperature of Deflection under Load—Part 1: Plastics and Ebonite.

[48] International Standards Organization (2011). ISO 178:2011—Plastics—Determination of Flexural Properties.

[49] International Standards Organization (2003). ISO 868:2003—Plastics and Ebonite—Determination of Indentation Hardness by Means of a Durometer (Shore Hardness).

[50] Weatherall I.L., Coombs B.D. (1992). Skin Color Measurements in Terms of CIELAB Color Space Values. J. Investig. Dermatol..

[51] Pawlak F., Aldas M., López-Martínez J., Samper M.D. (2019). Effect of Different Compatibilizers on Injection-Molded Green Fiber-Reinforced Polymers Based on Poly(Lactic Acid)-Maleinized Linseed Oil System and Sheep Wool. Polymers.

[52] Fox J. (2008). Analysis of Polymer Additives in the Packaging Industry.

[53] Liu G., Wu G., Chen J., Kong Z. (2016). Synthesis, Modification and Properties of Rosin-Based Non-Isocyanate Polyurethanes Coatings. Prog. Org. Coat..

[54] Wong R.B.K., Lelievre J. (1981). Viscoelastic Behaviour of Wheat Starch Pastes. Rheol. Acta.

[55] Costakis W.J., Rueschhoff L.M., Diaz-Cano A.I., Youngblood J.P., Trice R.W. (2016). Additive Manufacturing of Boron Carbide via Continuous Filament Direct Ink Writing of Aqueous Ceramic Suspensions. J. Eur. Ceram. Soc..

[56] Aldas M., Ferri J.M., Lopez-Martinez J., Samper M.D., Arrieta M.P. (2019). Effect of Pine Resin Derivatives on the Structural, Thermal, and Mechanical Properties of Mater-Bi Type Bioplastic. J. Appl. Polym. Sci..

[57] Redfern J.P.A.C. (1963). Thermogravimetric Analysis. Analys.

[58] Mamun A., Okui N. (2014). Miscibility and Thermal Studies of Isotactic Polystyrene and Poly (Cyclohexyl Methacrylate) Blends. Int. J. Nov. Res. Phys. Chem. Math..

[59] Gooch J.W. (1972). Encyclopedic Dictionary of Polymers.

[60] Wypych G. (2017). Effect of Plasticizers on Properties of Plasticized Materials: Effect of Plasticizers on Other Properties. Handbook of Plasticizers.

[61] Silvestre A.J.D.D., Gandini A. (2008). Rosin: Major Sources, Properties and Applications. Monomers, Polymers and Composites from Renewable resources.

[62] Eshraghi S., Das S. (2010). Mechanical and Microstructural Properties of Polycaprolactone Scaffolds with One-Dimensional, Two-Dimensional, and Three-Dimensional Orthogonally Oriented Porous Architectures Produced by Selective Laser Sintering. Acta Biomater..

[63] Jindal R., Sharma R., Maiti M., Kaur A., Sharma P., Mishra V., Jana A.K. (2017). Synthesis and Characterization of Novel Reduced Gum Rosin-Acrylamide Copolymer-Based Nanogel and Their Investigation for Antibacterial Activity. Polym. Bull..

[64] Elzein T., Nasser-Eddine M., Delaite C., Bistac S., Dumas P. (2004). FTIR Study of Polycaprolactone Chain Organization at Interfaces. J. Colloid Interface Sci..

[65] Amin M., Putra N., Kosasih E.A., Prawiro E., Luanto R.A., Mahlia T.M.I. (2017). Thermal Properties of Beeswax/Graphene Phase Change Material as Energy Storage for Building Applications. Appl. Therm. Eng..

[66] Aldas M., Rayón E., López-Martínez J., Arrieta M.P. (2020). A Deeper Microscopic Study of the Interaction between Gum Rosin Derivatives and a Mater-Bi Type Bioplastic. Polymers.

[67] Vasile C., Stoleru E., Darie-Niţa R.N., Dumitriu R.P., Pamfil D., Tarţau L. (2019). Biocompatible Materials Based on Plasticized Poly(Lactic Acid), Chitosan and Rosemary Ethanolic Extract I. Effect of Chitosan on the Properties of Plasticized Poly(Lactic Acid) Materials. Polymers.

[68] Fabra M.J., Jiménez A., Ataré L., Talens P., Chiralt A. (2009). Effect of Fatty Acids and Beeswax Addition on Properties of Sodium Caseinate Dispersions and Films. Biomacromolecules.

[69] Fabra M.J., Talens P., Chiralt A. (2009). Microstructure and Optical Properties of Sodium Caseinate Films Containing Oleic Acid–Beeswax Mixtures. Food Hydrocoll..

[70] Vogler E.A. (1998). Structure and Reactivity of Water at Biomaterial Surfaces. Adv. Colloid Interface Sci..

[71] Arrieta M.P., Peltzer M.A., López J., del Carmen Garrigós M., Valente A.J.M., Jiménez A. (2014). Functional Properties of Sodium and Calcium Caseinate Antimicrobial Active Films Containing Carvacrol. J. Food Eng..

[72] Hambleton A., Fabra M.J., Debeaufort F., Dury-Brun C., Voilley A. (2009). Interface and Aroma Barrier Properties of Iota-Carrageenan Emulsion-Based Films Used for Encapsulation of Active Food Compounds. J. Food Eng..

